# Mangrove restoration and coastal flood adaptation: A global perspective on the potential for hybrid coastal defenses

**DOI:** 10.1073/pnas.2510980123

**Published:** 2026-01-20

**Authors:** Timothy Tiggeloven, Vincent van Zelst, Eric Mortensen, Bregje K. van Wesenbeeck, Thomas A. Worthington, Mark Spalding, Hans de Moel, Philip J. Ward

**Affiliations:** ^a^Water and Climate Risk Department, Institute for Environmental Studies, Vrije Universiteit Amsterdam, Amsterdam 1081 HV, The Netherlands; ^b^CMCC Foundation - Euro-Mediterranean Center on Climate Change, Venice 30175, Italy; ^c^Deltares, Delft 2600 MH, The Netherlands; ^d^Department of Hydraulic Engineering, Faculty of Civil Engineering and Geosciences, Delft University of Technology, Delft 2600 GA, The Netherlands; ^e^Department of Plant Sciences, University of Cambridge, Cambridge CB2 3EA, United Kingdom; ^f^The Nature Conservancy, Siena 53100, Italy

**Keywords:** coastal flooding, nature-based solutions, climate adaptation, mangroves

## Abstract

To reduce current and future coastal flood risk, it is critical to better understand how adaptation measures, including nature-based solutions, can reduce that risk. Globally, hybrid coastal defenses, including a combination of coastal vegetation, such as salt marshes and mangroves, with a dike or sea wall, have been highlighted as a promising adaptation measure. Here, we present a global-scale assessment of the potential risk reduction from mangrove restoration in combination with foreshore dike systems under scenarios of climate and socioeconomic change. We provide a quantitative assessment of the benefits in terms of reduced economic damage, exposed population, and poverty exposure. We evaluate mangrove restoration fronting dikes by accounting for wave–vegetation interaction. If mangrove foreshore dike systems were established along coastlines susceptible to flooding, restoration could potentially reduce expected annual damage by US$800 million and reduce expected affected population by 140,000 annually. These values increase under future projections. Our benefit–cost analysis finds mangrove restoration economically viable for about half of the subnational regions assessed (85 to 105 out of 208). At the global scale, the benefit–cost ratio under future conditions ranges from 3 to 6, with a net present value between US$44 billion and US$125 billion. Because absolute risk values and benefit–cost analysis do not differentiate between relative wealth impacts, we also estimated restoration impacts across different wealth levels. We show that restoring mangroves disproportionately benefits people with lower incomes, as they are often more exposed to coastal flooding and located in areas suitable for mangrove restoration. As such, mangrove restoration in low- and middle-income countries could contribute to the resilience of people in poverty.

## Significance

This global assessment demonstrates that mangrove restoration presents a cost-effective nature-based solution for mitigating coastal flood risk for hybrid coastal defenses, offering substantial benefits in terms of reduced expected annual damage to infrastructure and population exposed to flooding. Crucially, this study underscores how mangrove restoration predominantly benefits vulnerable low-income communities, indicating its potential to reduce flood risk for poor populations and enhance resilience, particularly in low- and middle-income countries facing heightened flood risks.

In the coming century, coastal areas and their populations are projected to face increased flood risk driven by socioeconomic pressures and climate change (1–6). Climate shocks may exacerbate inequality and catalyze the formation of poverty traps in lower-income countries (7, 8), with coastal flood risk at the forefront of potential climate-driven impacts. To mitigate these impacts, it is critical to better understand both the flood risks and the outcomes of adaptation measures. Foreshore vegetation can play a significant role in protecting coastal areas from flood impacts because the presence and structure of vegetation dissipates energy from waves (9) and storm surges (10). For instance, during Hurricane Irma alone, mangroves are estimated to have averted US$1.5 billion in storm surge-related property damages in Florida (11). While attempts to quantify global flood protection benefits of mangrove forests have produced substantial valuations (12, 13), their precise economic value remains subject to considerable uncertainty due to methodological challenges. Incorporating foreshore vegetation in flood defense infrastructure alongside engineered defenses can result in a more sustainable and financially viable adaptation strategy compared to gray infrastructure alone (14, 15). These so-called hybrid coastal defenses have been highlighted as promising intervention in recent studies for their ability to provide adaptive, sustainable protection (16) while substantially reducing infrastructure costs through decreased design requirements (17).

Yet, large areas of mangrove forests have been lost or degraded due to human activities (18–21). The main driver of these losses is conversion to agriculture or aquaculture (21, 22). Over 3.4% of mangrove forests were lost in the 24 y leading to 2020, with considerably higher losses identified prior to that (23). Although loss rates have slowed considerably in recent years, future climate change and socioeconomic development will continue to threaten coastal ecosystems (24–27). The loss and degradation of these ecosystems disproportionally affects vulnerable communities that live close to the coast and that depend on these ecosystems for natural resources (28, 29). A particular example is constituted by local small-scale fishers, who often belong to the poorest groups in low-income countries, and depend on marine resources for subsistence and livelihoods (30–32). Increases in coastal flood risk due to sea-level rise and increased storminess, in combination with the loss of coastal ecosystems, can lead to poverty traps where people reliant on these ecosystems are disproportionately impacted (8, 33, 34).

In addition to reducing current flood risk, mangroves offer adaptive potential for mitigating future increased flood risk associated with climate change (35–37). Landward mangrove migration (38) combined with vertical accretion of coastal sediments within mangrove ecosystems at rates sufficient to keep pace with sea-level rise (39) can offer the potential to both mitigate climate impacts through carbon sequestration and enable coastal communities to adapt to changing environmental conditions (40–42). Many of the benefits typically associated with mangrove forests, such as carbon storage and fisheries enhancement, can be reinstated through mangrove restoration. It is therefore imperative to conserve remaining mangroves and identify those areas with the potential to be restored (43). However, most studies that assess the effectiveness of mangrove restoration in reducing current and future coastal flood risk are large-scale and low-resolution models, which cannot fully capture all of the factors that influence these benefits at the local scale (44).

Interestingly, ecosystem restoration may also have added benefits in terms of poverty reduction (45). Integrating flood risk and adaptation to climate change into conservation planning can therefore help direct investments in areas which specifically benefit vulnerable groups (46). This targeted approach ensures that adaptation measures not only address environmental goals but also contribute to social equity and economic resilience, creating a positive feedback loop between ecosystem health and human well-being in coastal communities facing climate threats.

This study provides a global-scale assessment of the potential flood risk reduction benefits of mangrove restoration for hybrid coastal defenses, including incorporation of future predictions of climate change and socioeconomic development. This research highlights regions where mangrove restoration could have the greatest flood mitigation benefit and provides a roadmap to target local-scale research. We extend the methodology developed by Tiggeloven et al. (15) assessing reduction in coastal flood risk assuming vegetated-foreshore dike systems by including potential mangrove restoration areas in front of these dikes in the modeling framework with assumed protection levels from the validated coastal FLOPROS database (4). Using the wave attenuation and dike overtopping model of van Zelst et al. (14) alongside the mangrove restoration potential model from Worthington and Spalding (43), we estimate current and future flood protection levels with and without restoration. A benefit–cost analysis, including implementation, maintenance, and opportunity costs but excluding co-benefits, is performed for mangrove restoration under climate and socioeconomic change, and finally a wealth disparity analysis is assessed for countries with high mangrove restoration potential.

## Results

### Global-Scale Flood Risk Benefits of Mangrove Restoration.

**Fig. 1.**
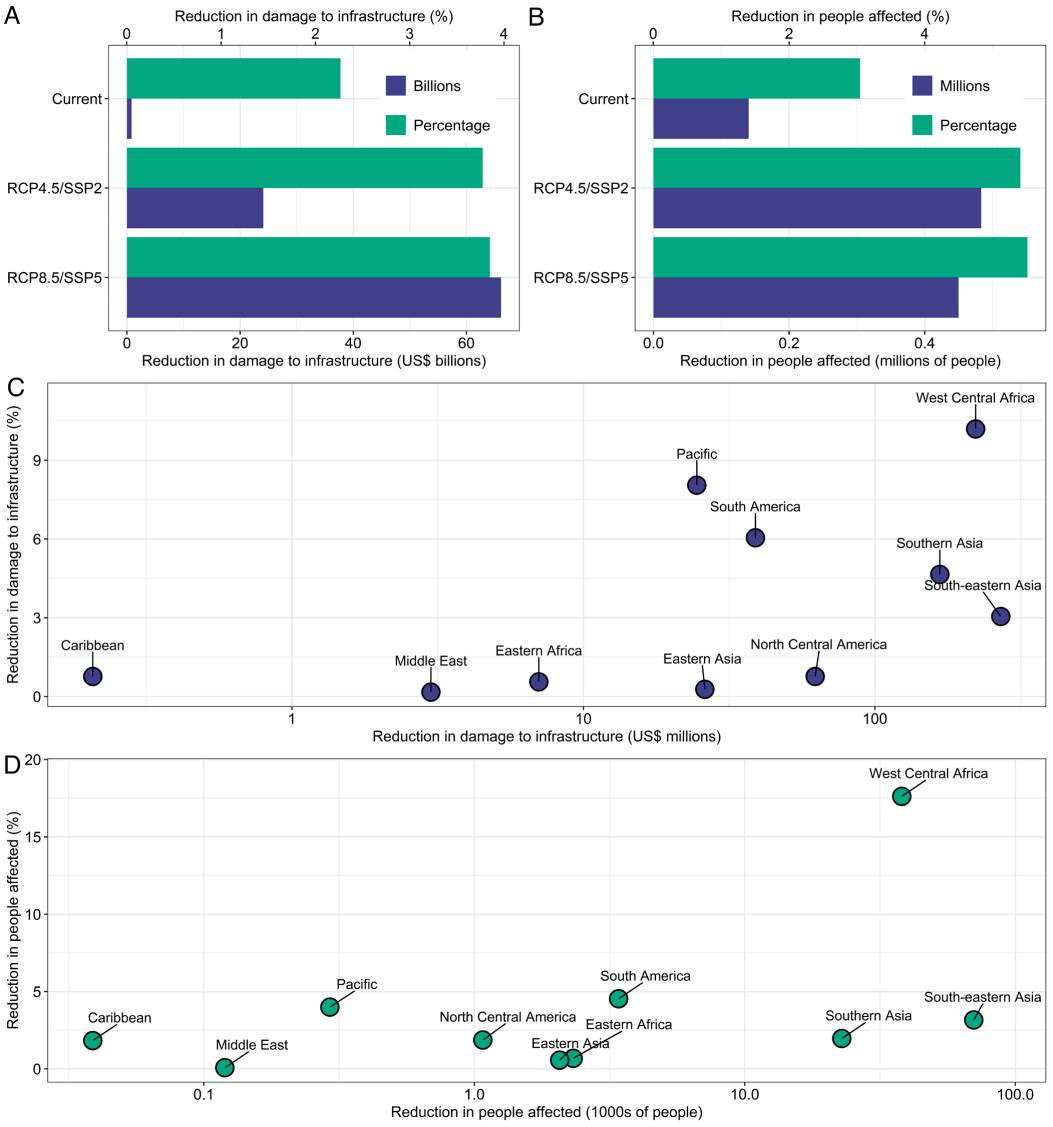
Flood risk reduction benefits of mangrove restoration under present-day conditions in terms of potential risk reduction in expected annual damage (EAD) to built-up assets, potential risk reduction in expected annual population affected (EAAP), and increase in protection levels. Panels *A* and *B* show global results for EAD and EAAP, and Panels *C* and *D* show regional results for EAD and EAAP.

Using flood risk models that incorporate rare extreme events, we developed estimates of the annual benefits provided by mangrove restoration in terms of damage reduction and population affected by inundation for present and future scenarios. If all potentially restorable mangrove areas are restored, under present-day conditions expected annual damage (EAD) to built-up assets caused by overtopping of coastal defenses linked to storm surge and wave-related flooding could potentially be reduced by US$800 million per year, which is equivalent to 2.3% of total damage projected for mangrove coastlines (Fig. 1). In addition, the number of expected annual affected population (EAAP) by flood risk can be reduced by 140,000 (3%). Although this represents a substantial benefit from reduction in coastal flooding impacts, the residual impact remains high. Under future climate and socioeconomic projections, the risk from storm surge and wave related flooding is significantly increased. Therefore, the total potential impact reduction as a result of restoration increases considerably relative to the present day for both EAD (30 to 80 times) and population affected (~12 times); however, as proportion of total impacts the reductions remain similar to current levels (3.8% and 5.4%, respectively).

### Regional- and National-Scale Flood Risk Benefits of Mangrove Restoration.

Southeast Asia has the highest absolute benefits from restoration, with more than two-thirds of the global potential risk reduction for both EAD (US$270 million per year) and reduction in population affected (70,000 per year) (Fig. 1). While West Africa ranks second in estimated absolute benefits (US$221 million; 38,000 per year), it is the region where the reduced flooding impacts represents the highest proportion of the total impacts for both EAD (11%) and expected annual affected people (18%). Similarly, South Asia also shows high potential reductions in EAD (US$167 million) and people affected (23,000 per year).

Among countries with documented coastal protection infrastructure [based on FLOPROS database validation (4)], we find the highest absolute potential risk reduction in terms of EAD under current conditions for India, Vietnam, and China (US$170, US$29, and US$26 million per year, respectively). For reduction in people affected, we find values of 21,000, 14,000, and 2,100 people per year, for the same countries, respectively.

Across all countries, we find that the highest absolute potential risk reduction in terms of EAD are in Nigeria, India, and Indonesia (US$0.2, US$0.17, and US$0.16 billion per year, respectively), while for reductions in EAAP it was largely the same countries: Nigeria, Indonesia, India, and the Philippines (35,000, 33,000, 21,000, and 17,000, respectively) (*SI Appendix*, Table S1). At the subnational scale, the distribution of these values is highly variable, with national benefits often largely driven by single subnational regions, such as Rivers State in Nigeria (which includes the extensive Niger Delta) and Maharashtra in India.

**Fig. 2.**
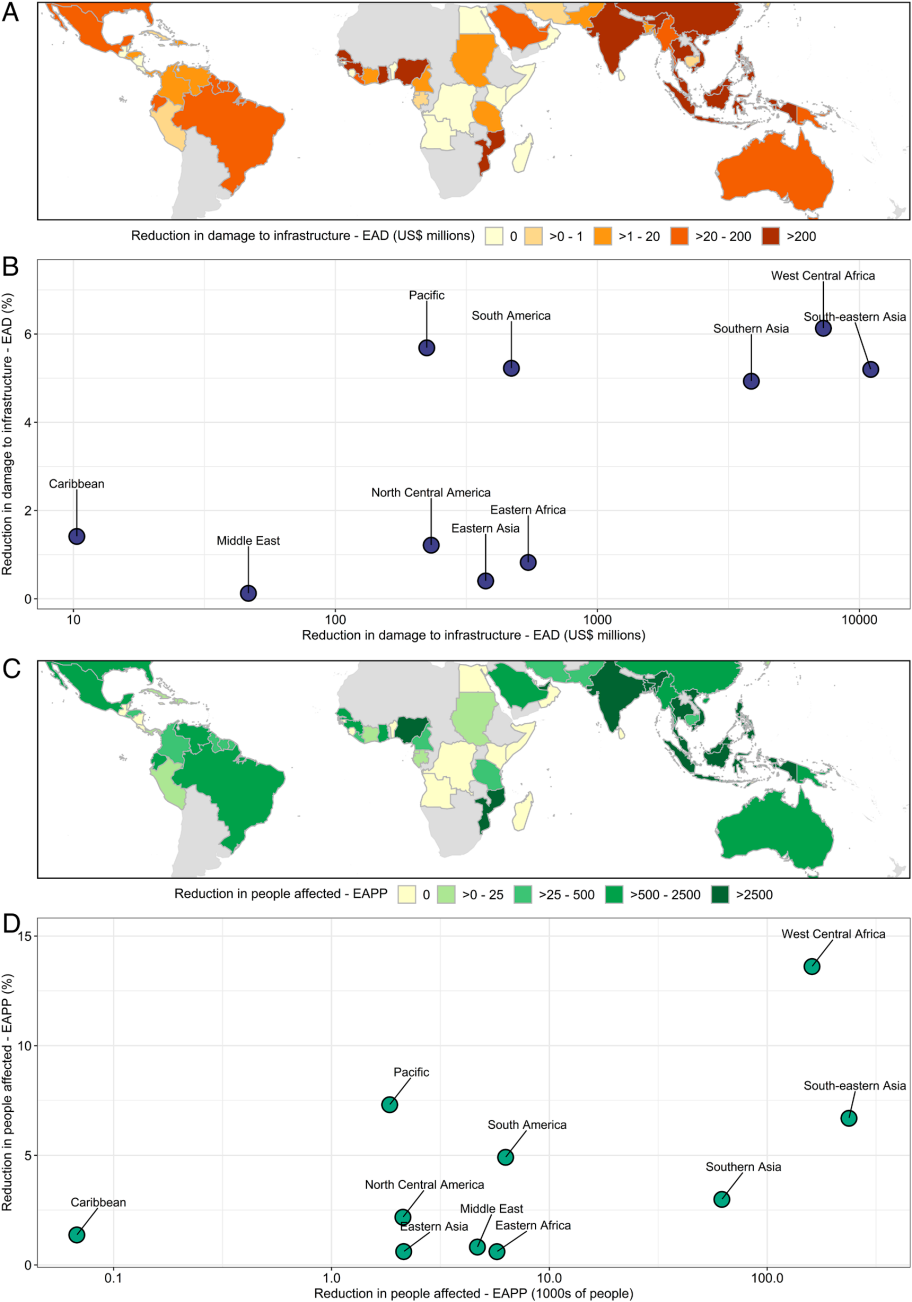
Future (2080) expected annual damage (EAD) reduction to built-up assets (*A* and *B*) and expected annual affected population (EAAP) (*C* and *D*) for countries (*A* and *D*) and supranational regions (*B* and *D*) under the scenarios RCP4.5/SSP2.

Under scenarios of socioeconomic and climate change, the highest potential reductions in future flood risk due to mangrove restoration are again centered in Asia and parts of West Africa (Fig. 2). Nationally, the countries with the highest projected reductions in future EAD are Nigeria (US$5.6 billion), Vietnam (US$4.5 billion), Indonesia (US$4.3 billion), and India (US$3.8 billion). It should be noted that these numbers are 2080 values, linked to projected future GDP values and that, as a proportion of GDP, they remain similar to present annual values. For EAAP, the highest projected reductions are Nigeria (150,000), Vietnam (97,000), the Philippines (76,000), and India (57,000). The potential benefits of mangrove restoration are highest in areas with large amounts of projected population growth. As with scenarios of current conditions, the value of impact reduction is highly variable at subnational levels, with numbers often concentrated in just a few smaller regions with relatively high population densities, such as Java (Indonesia), and Rivers State (Nigeria).

Locations with the greatest reductions in expected annual damages relative to the area of restorable mangrove are located in South Asia, East Asia, and Southeast Asia (*SI Appendix*, Fig. S1). These do not always co-occur with areas that are most effective in terms of population affected (which include, for example, regions in the Americas).

### Benefit–Cost Analysis of Mangrove Restoration.

**Fig. 3.**
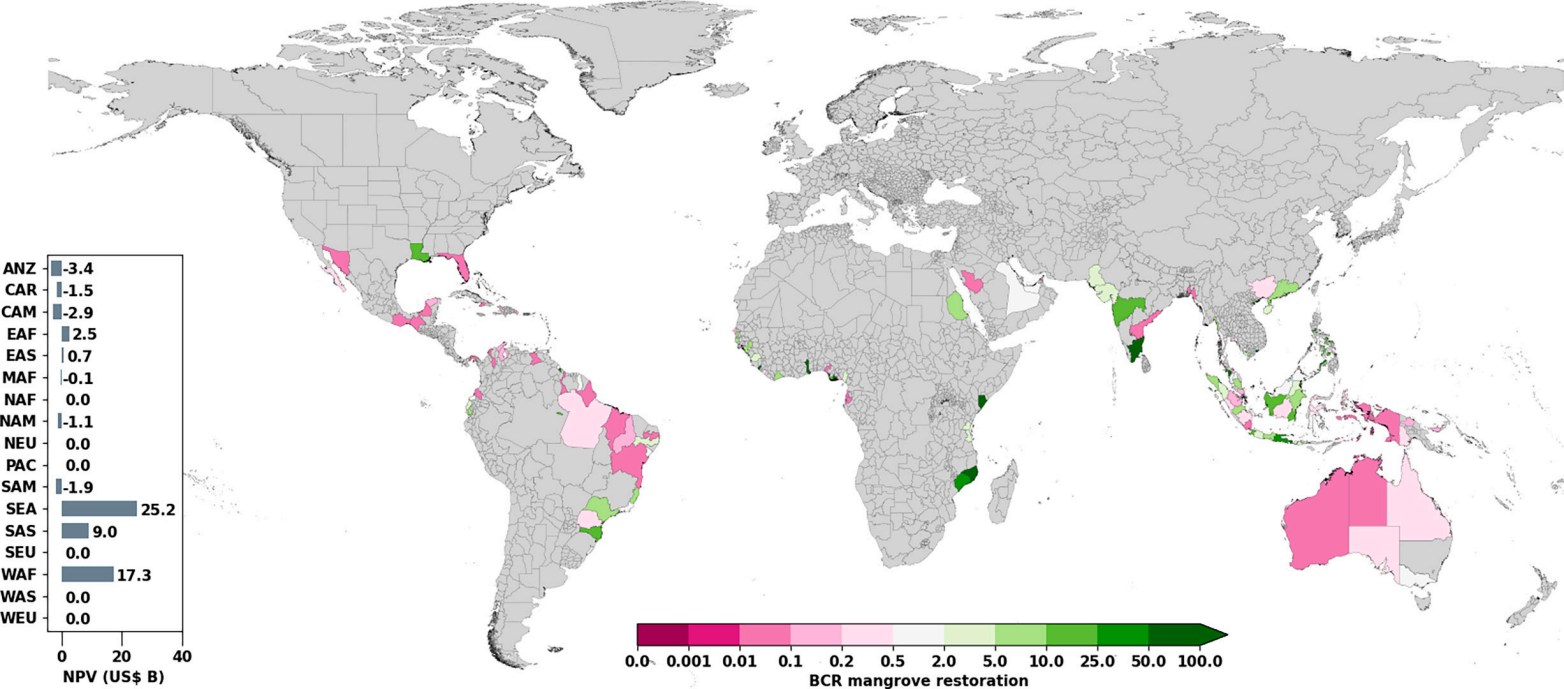
Benefit–cost ratios of mangrove restoration under the future scenario of RCP4.5/SSP2 shown for subnational regions in the world plot and subcontinental regions in the subplot. Regions that show up in gray are indicated to have no intersection of mangrove restoration, coastal flooding, and/or exposure. ANZ, Australia and New Zealand; CAR, Caribbean; CAM, Central America; EAF, Eastern Africa; EAS, Eastern Asia; MAF, Middle Africa; NAF, Northern Africa; NEU, Northern Europe; PAC, Pacific regions that include Melanesia, Polynesia, and Micronesia; SAM, South America; SEA, South-eastern Asia; SAS, Southern Asia; WAF, Western Africa; WAS, Western Asia; WEU, Western Europe.

Overall, mangrove restoration is estimated to be economically feasible, with a high benefit–cost ratio (BCR) between 3 and 6, and a net present value between US$44 billion and US$125 billion, depending on the RCP/SSP scenario. These BCR values are excluding co-benefits and costs beyond implementation, opportunity, and maintenance costs. We found BCR values greater than one estimated for almost half of the subnational regions assessed (41%; 85 out of 208) for RCP4.5/SSP2 (Fig. 3) and more than half (50%; 105 out of 208) for RCP8.5/SSP5 with a survival rate of 75% and a discount rate of 5% (*SI Appendix*, Fig. S2). Accounting for these benefits and costs through time (to 2100), net present value (NPV) estimates for restored areas are highest in Southeast Asia (US$25 billion), South Asia (US$9 billion), and West Africa (US$17 billion), with India, Indonesia, Vietnam, and Nigeria (*SI Appendix*, Table S1) having the highest national NPV. In a sensitivity analysis to various discount rates, survival rates, and exclusion of opportunity costs, we see consistently high BCRs for regions in Eastern Africa, Eastern Asia, Southeastern Asia, South Asia, and West Africa (*SI Appendix*, Table S2). In this sensitivity analysis and at the global scale only one run out of twelve returned BCR<1. In the sensitivity analysis for RCP4.5/SSP2, we find that BCR ranges between 0.8 and 5.3, NPV ranges between US$-3.6 billion and US$130 billion, and the number of regions with positive BCR ranges between 57 and 102. For RCP8.5/SSP5, BCR ranges between 1.3 and 12.6, NPV ranges between US$6.5 billion and US$136 billion, and the number of regions with positive BCR ranges between 63 and 124.

Most of the subnational regions with a projected negative return on investment are located in Central America, South America, and the Caribbean, as the assets and infrastructure being exposed to coastal flooding are relatively low in these regions in the model. The majority of the areas that show BCRs lower than or close to 1 have relatively low total discounted benefits. Other subnational regions with relatively high total discounted benefits (high percentage of assets), but low BCRs are mostly located in high-income countries such as Australia or the United States, as adaptation measures in these regions are more expensive due to higher prices for resources, labor and opportunity costs. Note that our benefit–cost analysis does not include co-benefits and evaluates full restoration potential at aggregate scales, which may mask site-specific opportunities with positive returns in regions showing negative average benefit–cost ratios.

### Mangrove Restoration and Poverty.

A large proportion of the people living in flood-prone areas are also living in poverty: 75% (26 million people) in Bangladesh; 67% (300,000 people) in Nigeria; and 87% (80,000 people) in Pakistan (Table 1). For Pakistan this proportion is much higher than the country as a whole (87% vs. 51%); in Bangladesh this proportion is similar to the national value, whereas in Nigeria, it is lower than the rest of the country (67% vs. 74%), as Nigeria has most of large city hubs located at the coast. These trends are similar for the proportion of population living in areas where mangrove restoration is feasible. While total numbers of people living in poverty are expected to increase by the year 2050, the proportion of those living in flood-prone areas or areas where mangrove restoration is possible remains stable.

**Table 1.** Overview of the percentage of people living in poverty for the whole population, living in flood-prone areas, and living in flood-prone areas where mangrove restoration is possible

		Country population		Flood-prone population		Population in areas where mangrove restoration is possible	
Country	Time	In poverty	Total	In poverty	Total	In poverty	Total
Bangladesh	Present-day	114 (76%)	149	26 (75%)	34	11 (75%)	15
	2050	196 (76%)	150	41 (74%)	56	14 (75%)	19
Nigeria	Present-day	117 (74%)	158	0.3 (67%)	0.4	0.3 (67%)	0.4
	2050	277 (75%)	370	1.6 (66%)	2.5	1.6 (66%)	2.5
Pakistan	Present-day	88 (51%)	173	0.08 (87%)	0.1	0.08 (88%)	0.09
	2050	151 (52%)	292	0.16 (81%)	0.2	0.16 (81%)	0.2

Numbers are displayed in millions.

**Fig. 4.**
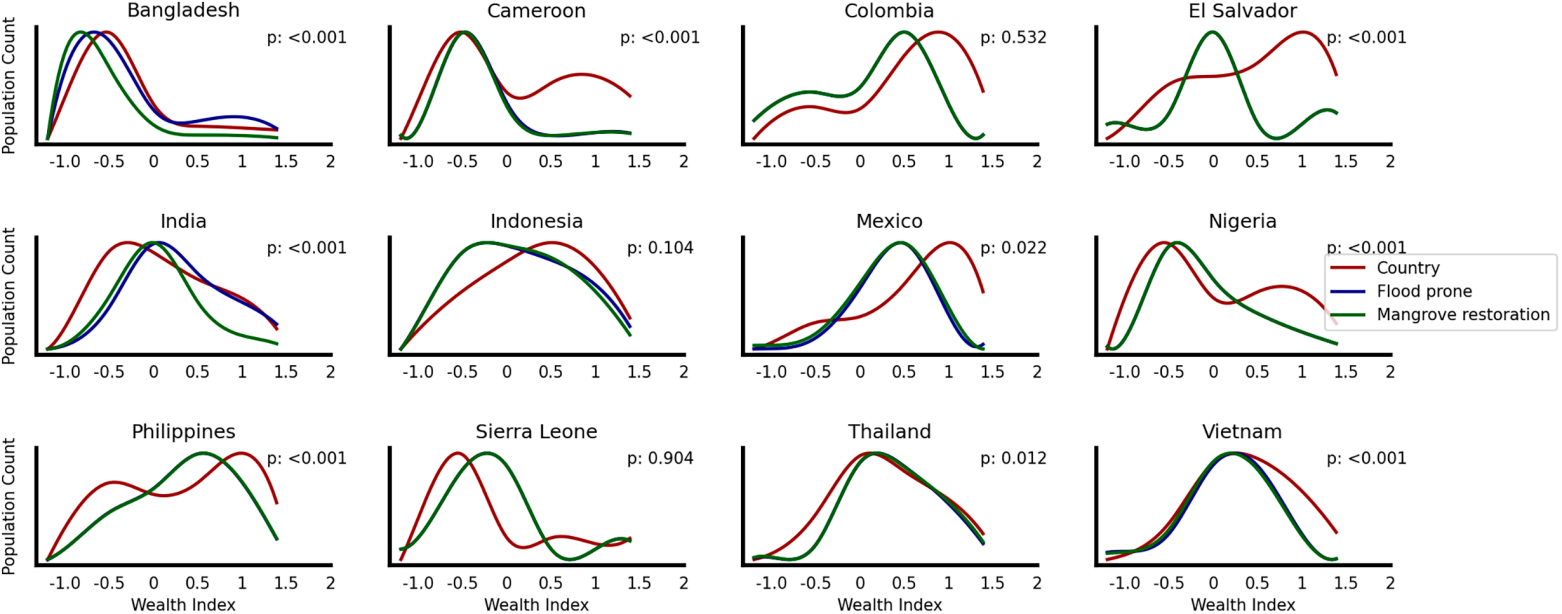
Normalized distribution of the wealth index for people living outside flood-prone areas (red line), inside flood-prone areas (blue line), and potential mangrove restoration areas (green line). On the x-axis the global wealth index where 0 is the country mean and on the y-axis the normalized population count. Note that for all cases where the blue curve is not visible, it is overlapping with the green curve.

The asset-based wealth distribution in areas with potential mangrove restoration differs significantly from regions without such potential. Fig. 4 illustrates the disparity in wealth, depicting shifts in population curves for those living outside flood-prone areas (red), inside flood-prone areas (blue), and inside flood-prone areas with potential mangrove benefits (green). In several countries, including Bangladesh, El Salvador, Mexico, Indonesia, and the Philippines, populations in non-flood-prone areas tend to be wealthier. Notably, in Bangladesh and Cameroon, higher wealth index individuals (>0) are concentrated in regions where mangrove restoration is not feasible. The same pattern of higher wealth index (0 and above) is more likely to reside outside flood-prone areas and is found in Cameroon, Colombia, El Salvador, Indonesia, Mexico, Nigeria, and Vietnam. While mangrove restoration may not be economically viable for El Salvador, considerations of equity in adaptation strategies reveal potential benefits for individuals with lower wealth indices in the country. Although there is variation between countries, overall, the results suggests that mangrove restoration as an adaptation measure favors populations with lower wealth levels, and as such could contribute to more equitable risk distributions and address inequality. This benefit distribution is particularly pronounced across the urban–rural divide, where rural coastal communities with fewer engineered protection measures and greater dependence on natural resources stand to gain substantially more from such nature-based solutions (47).

## Discussion

This work provides a global-scale assessment of the impact of mangrove restoration on current and future potential flood risk reduction for hybrid coastal defenses under scenarios of socioeconomic and climate change. We show that mangrove restoration can play an important role in climate change adaptation especially benefitting low-income groups. We also demonstrate that the future benefits of mangrove restoration can be assessed using existing flood risk assessment frameworks. This study indicates that mangroves can be efficient and cost-effective in reducing risk for many regions, with the greatest benefits estimated for parts of Asia and West Africa. Our findings are in line with other studies on risk reduction by coastal ecosystems, such as (14, 15, 48–51), with existing mangroves estimated to reduce present-day damages by 9 to 13% across subnational regions of the world where they are extant (12) (15). By implementing comprehensive mangrove restoration worldwide, EAD could be reduced by an additional US$0.8 billion annually. These potential global benefits of mangrove restoration align with evidence at the local scale (52). For instance, in Bangladesh, leveraging existing mangrove belts to reduce wave impacts potentially allows for reduction of embankment revetments with mangrove ecosystems (17), which demonstrates the economic advantages of hybrid coastal defense systems.

This study shows that the effectiveness of mangrove ecosystems in mitigating flood risks becomes even more pronounced with future climate scenarios. Climate change, and particularly sea-level rise, will influence the potential for mangroves to serve as natural barriers against storm surges and coastal flooding (20, 53). Understanding their effectiveness under different climate scenarios is imperative for informed decision-making (54). Our findings highlight the ongoing role that restored mangroves fronting dikes could play in coastal defense, further supporting arguments for restoration in the face of coastal change. Policymakers and environmental practitioners can use these findings to prioritize and guide mangrove restoration projects at regional and national scales, though site-specific assessments remain necessary. Several challenges, however, may arise, such as coastal squeeze and relative sea-level rise (55–57). Additionally, the dynamic nature of climate change introduces uncertainties in projecting the future effectiveness of mangrove restoration efforts (53, 58). Climate change can alter the conditions that make mangroves effective in flood risk reduction, making long-term planning and adaptability crucial components of restoration strategies.

Based on our projected benefit–cost ratios, the financial viability of mangrove restoration globally implemented appears high, albeit with a large range of uncertainty (BCR between 3 and 6 for RCP4.5/SSP2 and RCP8.5/SSP5 respectively) driven by the range of potential future climate and socioeconomic change scenarios. Further challenges not adequately described in BCR estimates, arise from temporal and socioeconomic challenges. BCR values are based on projections from subnational regions where the costs and benefits from restoration are spent and gained by different social and economic groups. Furthermore, while the costs of investment are relatively short-term, the returns may be long-term and linked to rare, high-impact events. These factors may create considerable barriers to investment.

We have provided a sensitivity analysis to BCRs taking different discount rates, survival rates, and the exclusion of opportunity costs and found that although BCRs range between 0.8 and 5.3 for RCP4.5/SSP2 and 1.3 and 12.6 for RCP8.5/SSP5. The only run model that generated a global BCR <1 was with a discount rate of 10% for RCP4.5/SSP2. However, at the regional scale, there are consistent positive BCRs in regions of Eastern Africa, Eastern Asia, Southeastern Asia, South Asia, and West Africa throughout all sensitivity runs. Survival rates represent a critical uncertainty in mangrove restoration. Many ambitious projects have been recorded as failing and, although there is now a strong body of literature supporting best practice restoration methods (59, 60), postrestoration monitoring remains poor (61). Despite these legitimate concerns, we show in our analysis that the economic model for restoration is more sensitive to varying discount rates (3, 5, 8, and 10%) than survival rates (50, 75, and 90%).

In addition to coastal protection, restoration has the potential to generate numerous additional benefits as nature contributions to people such as provision of timber and fuelwood, erosion control, reduced saltwater intrusion, water quality, enhanced fisheries, and increased biodiversity, as well as cultural services such as recreation (32, 62, 63). More comprehensive cost modeling at local scales would ideally incorporate other costs excluded in this study (e.g., transactional and land acquisition costs), but also include these co-benefits. The importance of mangroves in carbon storage and sequestration is another benefit receiving world-wide attention (64). These unique co-benefits remain insufficiently understood in their distributional impacts, yet emerging evidence suggests they may disproportionately benefit communities living in poverty who rely more directly on these ecosystem services for their livelihoods and well-being (47, 65). Beyond simple economic arguments, our use of poverty indicators also highlights further possible motivations for restoration, with the model predicting uneven benefits and the potential to disproportionately advantage those living in poverty. However, a more rigorous distributional analysis would require weighting avoided losses by marginal utility of income to capture the greater welfare value that flood protection provides to lower-income households and to better quantify the equity dimensions of mangrove restoration benefits.

Given the global scale of this analysis, several assumptions related to mangrove dynamics in vegetated foreshore-dike systems were required, and our input data were not of sufficient resolution to show finescale local patterns. At the local scale the selection of restoration sites is critical, and some locations will have a far greater impact on flood risk reduction than others. For example, the effectiveness of mangrove coastal protection depends on the forest width. The current analysis is based on wave attenuation by mangroves which is nonlinear. Adding small patches in extensive forests is therefore unlikely to yield notable benefits. Conversely, establishing continuous mangrove belts of sufficient width in currently unprotected areas or restoring degraded forests in high-exposure zones with vulnerable populations nearby would likely provide the most significant flood protection benefits and socioeconomic returns. Moreover, the work does not account for the variable ability of mangroves to maintain surface elevation through vertical accretion and assumes they maintain their elevation relative to sea level in future scenarios (39, 66). It also does not account for the natural dynamics of mangrove communities, which can lead to changes in distribution, particularly in areas of active sediment movements. Additionally, dikes can negatively impact sediment dynamics in mangrove greenbelt, due to wave reflection (67). Finally, the additional direct impacts of extreme conditions such as tropical cyclones, which can include dramatic changes in mangrove cover and elevation are not included in this study.

The modeling framework itself also has limitations. Mangrove coastlines are typically complex and convoluted, and as such are difficult to represent with simple shore-normal transects. In some model transects, existing mangroves or restorable areas may extend well beyond the 4 km transect length, while the spacing of the transects, means that some areas of potential mangrove restoration are likely omitted in our findings. In addition, lateral flood effects of surges are not taken into account under our transect approach. Local spatial forest configuration is also not included, generating large uncertainties about surge attenuation (68). Including a 2D approach for surge attenuation on global scales would improve global estimations of the total contribution of mangrove forests to inundation reduction. Finally, our analysis assumes the presence of functional dike systems based on FLOPROS protection standards. While this represents the only global model for such protection, and while it attempts to account for variability in build quality driven by income levels and urbanization, it does not represent reality (69). In addition, many mangrove restoration sites may lack any such engineered infrastructure or have limiting opportunity to combine such green-gray infrastructure approaches. Thus, our benefit estimates may over- or understate the marginal protective value of mangroves where dike specifications are incorrectly characterized.

In reality, achieving successful restoration outcomes is challenging, and is often restricted by societal, cultural, institutional, or economic barriers, as well as biophysical conditions (70). For example, studies show that most mangrove restoration projects fail primarily due to governance failures, planting in unsuitable locations, land tenure conflicts, lack of community engagement and institutional barriers rather than technical limitations (71–73). These implementation challenges, including transaction costs and systematic avoidance of suitable sites due to unresolved property rights may suggest that by ignoring institutional realities purely economic assessments may significantly overestimate restoration feasibility (70, 74). Despite its limitations, our analysis strengthens the body of evidence that mangrove restoration may play a crucial role in future climate adaptation. Future risk reduction in coastal areas will result from a combination of approaches, including engineering, societal change, and other adaptation strategies. For example, incorporating these hybrid adaptation strategies that use both gray and green infrastructure can further optimize flood mitigation strategies (14, 75).

While economic estimates can be powerful means to influence policy, they can show bias toward wealthy nations with high GDP. Alongside such metrics, our model also shows the considerable social benefits that would accrue in low- and middle-income countries. Implementing mangrove restoration in these countries, notably in Africa and Asia, can contribute to the resilience of people in poverty, support poverty alleviation and help tackle poverty traps.

Our findings lend strong support to current policy commitments and efforts calling for widescale restoration of habitats (e.g., the UN Decade of Restoration, https://www.decadeonrestoration.org/); of forests (the Bonn Challenge, https://www.bonnchallenge.org/); and specifically of mangroves [the Global Mangrove Alliance, (23)]. The education and training of practitioners and scientists, including those based in local communities, is vital to enhance understanding on the protection, conservation, and management of restored mangroves (76), as without strong support, restoration efforts are challenging and likely to fail (77). While considerably greater detail would be required to prioritize locations and approaches for restoration on the ground, the model outputs provide a clear demonstration of the logic of prioritizing restoration in locations worldwide to achieve long-term social and economic returns.

## Materials and Methods

**Fig. 5.**
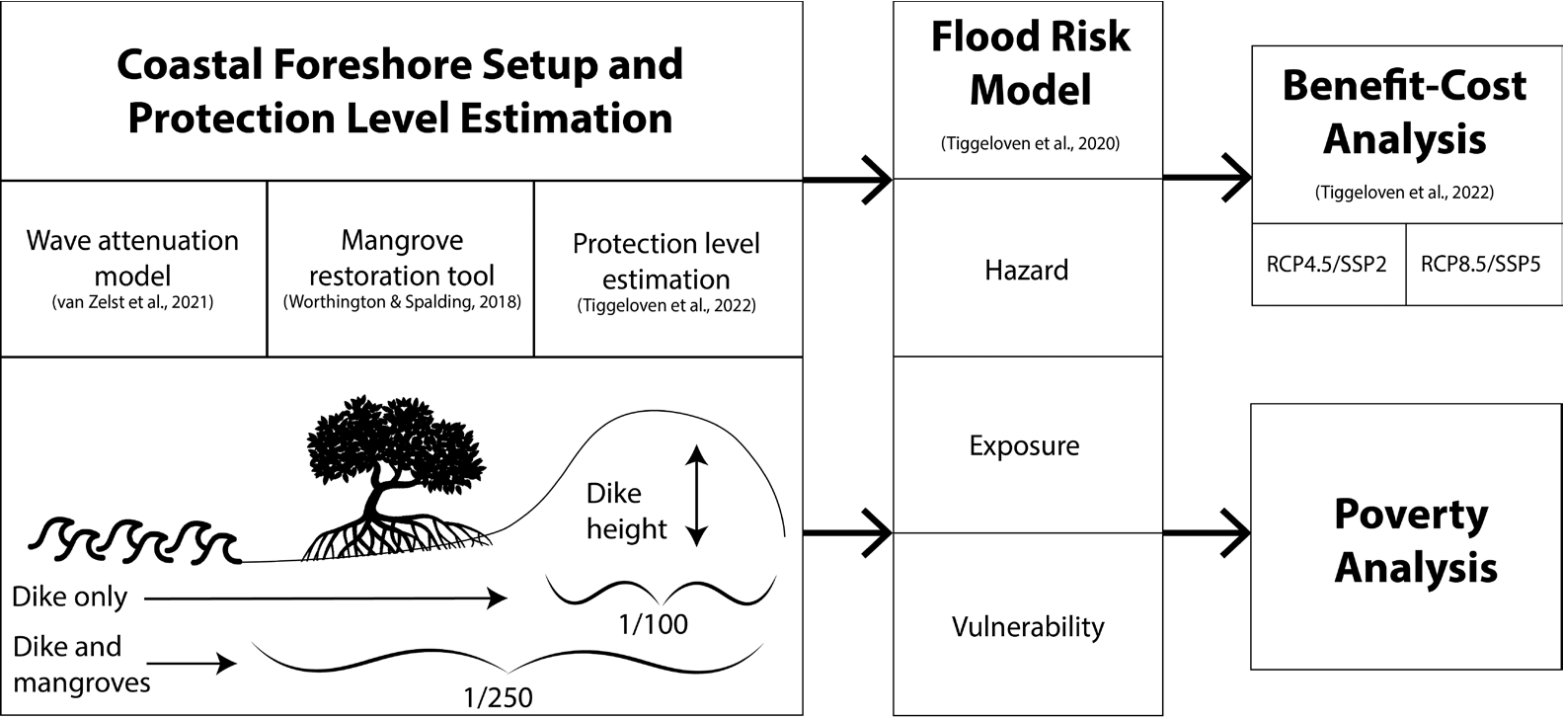
Flowchart of the methodology used in this study with an illustration of protection level estimation.

This study estimates global-scale flood protection benefits of mangrove restoration for hybrid coastal defenses under current conditions, and future socioeconomic and climate change scenarios. We extend on the methodology described in ref. 15, to assess reduction in coastal flood risk by including areas of potential mangrove restoration in the modeling framework. The analysis consists of the following main steps: 1) coastal foreshore setup and protection level estimation; 2) flood risk modeling; 3) benefit–cost analysis for socioeconomic and climate change scenarios; and 4) a wealth disparity analysis for countries with high mangrove restoration potential. In brief, we use the wave attenuation model of van Zelst et al. (14) and mangrove restoration potential analysis by Worthington and Spalding (43) to estimate current and future flood protection levels (Fig. 5) resulting from restoring areas of recent mangrove loss. Flood risk is estimated as a function of hazard, exposure, and vulnerability (78). The damage to built-up assets [expected annual damages (EAD)] and the people affected [Expected annual affected population (EAAP)] over time are calculated with and without mangrove restoration, with the difference between those two scenarios representing the benefits of restoration.

### Coastal Foreshore Setup and Protection Level Estimation.

**Fig. 6.**
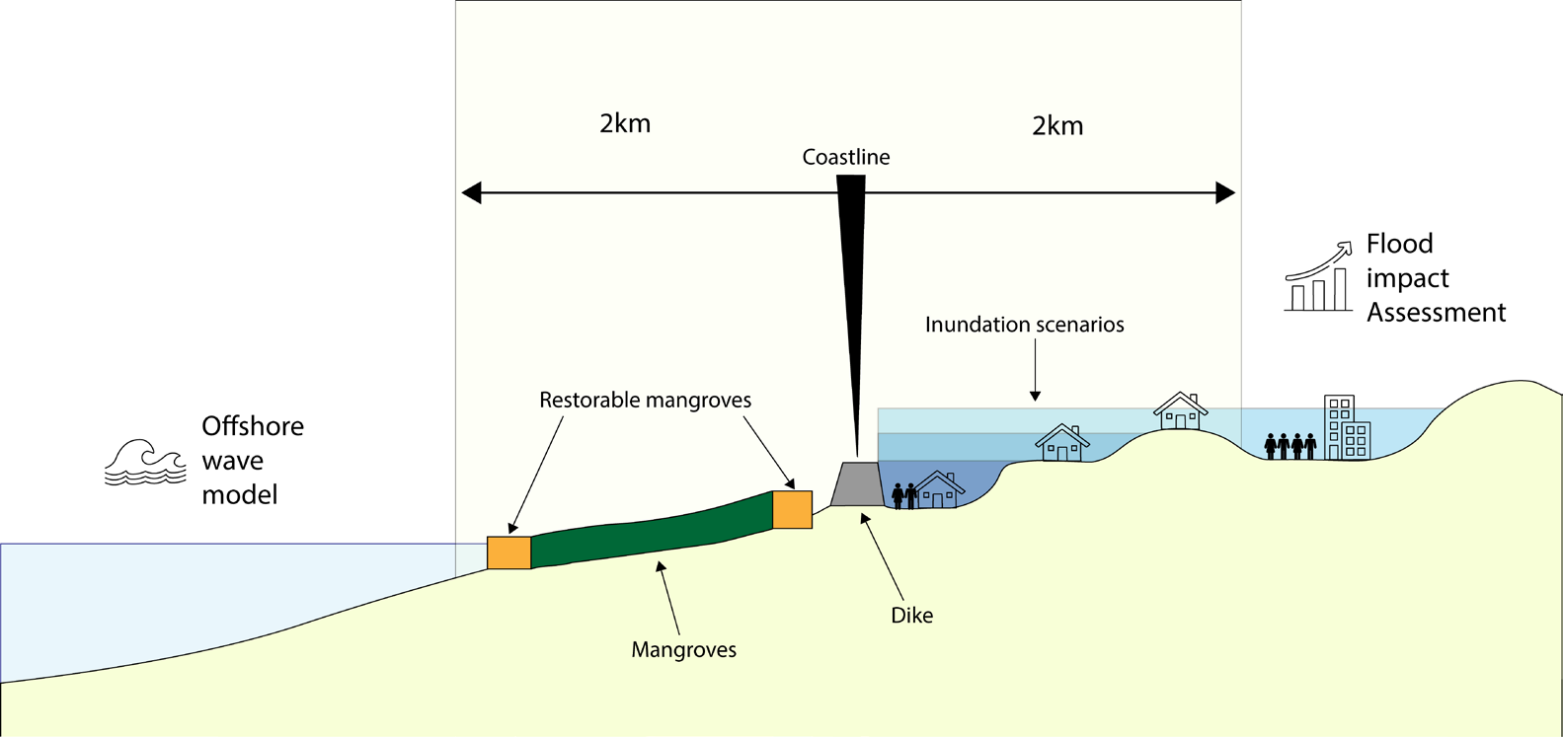
Schematic diagram showing a hypothetical shore-normal transect centered on a coastline. Beyond the transect, 2D data are used to inform the model both for offshore wave settings and landward risks to population and built-up assets.

The following section provides an overview of the coastal properties and wave attenuation model as developed by van Zelst et al. (14), used to quantify the wave–vegetation interactions. This model simulates attenuation of waves, on sea to coast trajectories, across 495,361 km shore-normal transects, using the transects’ depth, slope, and friction properties to support modules describing wave propagation and wave attenuation. The transects’ properties are described using data on water levels, elevation, waves, and vegetation presence. The model then incorporates an overtopping module to estimate required dike crest heights (see below). Wave attenuation (the dissipation of wave energy) is influenced by depth-induced wave-breaking and bottom friction and drag induced by the proportion of mangrove vegetation along the transect. The addition of more vegetation as a result of restoring mangrove areas on a transect allows the estimation of expected benefits from that restoration (Fig. 6).

Wave heights and water levels at the spatial start point of each transect are derived from a 2D offshore wave propagation model that sets the initial conditions for each transect. Wave propagation conditions per transect from the ERA-Interim global climate reanalysis dataset (79) were obtained using a peak-over-threshold approach for multiple return periods. Offshore significant wave heights were transformed to a nearshore wave height limited by depth-induced breaking. Water levels are derived from the GTSR dataset (80) and corresponding wave conditions at different return periods from the ERA-Interim reanalysis (79). To represent a worst-case scenario, wave angle of incidence is assumed to be coast normal.

Wave attenuation over the transect is determined using a lookup-table to estimate the resulting significant wave height relevant for the flood defense. This is done by combining 668,304 XBeach (81) hydrodynamic numerical modeling results for combinations of foreshore slopes, vegetation cover, and hydrodynamic conditions (14). This table contains wave heights at regular intervals along a steady slope, both with and without mangrove vegetation as modeled by XBeach. Present-day coastal flood protection, including the influence of extant mangroves, are taken from Tiggeloven et al. (4).

In our model, we assume that all mangrove areas are backed by dikes (a standard 1:3 dike profile without berms and an allowed overtopping discharge of 1 l/s/m). Dike heights required to prevent inland flooding per return period are theoretically estimated by assessing bed levels, hydrodynamic conditions, and wave attenuation for each transect. Overtopping is estimated using the empirical EuroTop formulations (82) with respect to the storm surge level. While this is clearly not everywhere the case, dikes are widely used in areas where mangroves are backed by human land uses. This is a more reliable and conservative estimate of likely protection in these areas. Current estimations of protection standards for the assumed dikes are taken from the FLOPROS database (4), with protection standards ranging from 2- to 1,000-y return periods depending on the country’s income level and urbanization, while accounting for the gradual degradation of these protection standards due to sea-level rise and subsidence in our flood risk projections.

To estimate the potential total area of mangrove restoration, and therefore contributions to existing protection standards, data on recent (1996 to 2016) mangrove loss were spatially intersected with the transects with the wave attenuation effects of mangrove restoration calculated using the same foreshore set-up. We projected restorable mangrove widths at 495,361 transects by taking a buffer of 1 km and calculating the mean mangrove width based on the transects in the buffer that are in a region that is estimated to have flood risk. Of a total of 6% restorable mangroves, 0.5% remains after this step. The mangrove widths without restoration are based on Global Mangrove Watch year 2010 (83). This results in increased protection levels due to the greater wave–vegetation interactions of the restored mangroves. An overview of distribution of mangrove restoration for urban and rural transects are shown in *SI Appendix*, Fig. S3. Subnational units that contain a potential area of mangrove restoration lower than 5 ha are excluded in this study to reduce data uncertainty.

### Flood Risk Estimation.

We combine coastal flood hazard layers with exposure data, and vulnerability curves to estimate the flood impacts by using the GLOFRIS module developed by Ward et al. (84), incorporating the future simulations of Winsemius et al. (85), and extended to coastal flood risk assessment by Tiggeloven et al. (4). The flood impacts are assessed at a horizontal resolution of 30 arcseconds (~1 km) and simulated for multiple return periods.

#### Flood hazard.

We project coastal water levels using hydrodynamic simulations of tide and surge. From these simulations, we derive sea levels for nine different return periods (2, 5, 10, 25, 50, 100, 250, 500, and 1,000 y). These return-period sea levels force a planar inundation model that estimates attenuation similar to Vafeidis et al. (86), and is described in detail by Tiggeloven et al. (4). Extreme sea-level values from the Global Tide and Surge Reanalysis (GTSR) dataset by Muis et al. (80) are taken to calculate inundation depths using the Multi-Error-Removed Improved-Terrain (MERIT) DEM (87) (30 arcseconds (~1 km) resolution) as the underlying topography. Because tropical cyclones are poorly represented in the climate input data of GTSR, we used an enriched version of GTSR that includes simulated tropical cyclones using the IBTrACS (International Best Track Archive for Climate Stewardship) archive, as described by Tiggeloven et al. (4). We apply a resistance factor to simulate the reduction of flooding inland as tides and storm surges have a limited time span.

In our future flood hazard simulations, we use projected sea-level rise to simulate extreme sea levels and land subsidence rates to estimate how the coastal topography may change. Global mean sea-level rise projections are obtained from the RISES-AM project (88) and regionalized using spatial variability associated with gravitational-rotational fingerprints (89). We use sea-level rise for two Representative Concentration Pathways (RCPs; RCP4.5 and RCP8.5), and include a range of probabilistic outcomes (5th, 50th, and 95th percentiles). Subsidence rates taken from the SUB-CR model by Erkens et al. (90) are more regionally variable, and for some regions exceed sea-level rise rates. Subsidence is modeled in response to groundwater extraction, which is the dominant factor of human-induced subsidence in many coastal areas (91, 92).

#### Flood exposure.

The methodology to derive flood exposure in terms of built-up area, population, and GDP are described in ref. 4. Built-up area represents all urban areas and artificial (built) surfaces. Current maximum economic damages are estimated using the methodology of Huizinga et al. (93). The approximate percentage area of different occupancy types per urban grid cells was set to 75% residential, 15% commercial, and 10% industrial, based on a study by the Buildings Performance Institute Europe (94) and a comparison of the European cities’ share of occupancy type of the CORINE Land Cover data (95). Following Tiggeloven et al. (4), the density of buildings per occupancy type is set to 20% for residential and 30% for commercial/industrial. Future simulations of built-up area are taken from Winsemius et al. (85) at a resolution of 30 arcseconds (~1 km). To estimate future maximum damages, we scale the current values with growth in GDP per capita per country from the Shared Socioeconomic Pathways (SSP 2 and 5) database. Boundaries of countries are derived from the Global Administrative areas dataset (96). To calculate future risk relative to GDP and population exposed, future gridded population and GDP values are taken from Huijstee et al. (97), which uses the national GDP per capita from the SSP database as input.

#### Flood vulnerability.

For each occupancy type different global flood depth-damage functions are used to estimate vulnerability to flood water in urban areas (93). Damages are represented as a percentage of the maximum damage, which reaches a maximum at an inundation depth of 6 m. Subsequently, flood impacts are calculated by estimating the percentage of maximum damage per occupancy type for the different inundation depths:

[1] $$I_{\theta }\left(w\right)=\theta_{r}\left(w\right)M_{r}+\theta_{c}\left(w\right)M_{c}+\theta_{i}\left(w\right)M_{i},$$

where $I_{\theta }$ is the flood impact at inundation depth of w, θ is the vulnerability at an inundation depth, and M is the maximum damage assigned for residential (r), commercial (c), and industrial (i) occupancy types.

#### Flood risk.

Expected annual damage to built-up assets (EAD) due to flood impacts per return period is estimated at the resolution of 30 arcseconds (~1 km). EAD is estimated by taking the integral of the exceedance probability-impact (risk) curve (98):

[2] $$D=\underset{p=0}{\int^{1}}I_{\theta }\left(p\right)dp,$$

where D is EAD, I is the urban damage (or impact) with θ representing the vulnerability, and p denotes the annual probability of nonexceedance. To fit a protection level of a coastal region in the risk computation, the risk curve is truncated at the exceedance probability of the protection level (expressed as a return period). To estimate the definite integral, we use the trapezoidal approximation. As data on coastal protection levels are not available for many regions, we estimate current protection levels using the FLOPROS modeling approach (99), which is described and validated for the coastal flood protection by Tiggeloven et al. (4). Using the same approach, people affected (EAAP) is estimated by replacing the damage with population exposed to a flood hazard. EAD and people affected are estimated with and without mangrove restoration.

### Benefit–Cost Analysis.

We perform a benefit–cost analysis and estimate the financial feasibility of restoration using two indicators, namely net present value (NPV), which is the net return on investment discounted to present value, and the benefit–cost ratio (BCR), which is the ratio between discounted benefits and discounted costs. The benefit of the investment in adaptation is the avoided damages expressed as the difference between EAD with and without mangrove restoration (Eq. **3**).

[3] $$B=\underset{p=0}{\int^{p=p_{n}}}I_{\theta }\left(p\right)dp-\underset{p=0}{\int^{p=p_{a}}}I_{\theta }\left(p\right)dp,$$

where B is the benefit of investment, $p_{n}$ is the annual probability of nonexceedance when no adaptation is implemented, and $p_{a}$ is the annual probability of nonexceedance when adaptation is implemented. The costs of mangrove restoration were derived from the spatially explicit cost model developed by Goto et al. (100), based on implementation costs from 249 restoration projects across 25 countries. This model estimates restoration costs as a function of project attributes including initial site condition (e.g., aquaculture ponds, deforested sites, highly eroded sites), geomorphic class (delta, estuary, lagoon, open coast), project size, and national GDP per capita. The model yields a global median implementation cost of US$8,143 per hectare (ranging from US$27 to US$253,300 per hectare across restoration sites), with maintenance costs set to 1% of implementation costs per year (35, 101–103). We used the nearest neighbor mangrove restoration cost value for each site in our projections. All costs were converted to US$ 2005 Power Purchasing Parity (PPP) by first adjusting to US$ 2005 values using GDP deflators from the World Bank Open Data website (https://data.worldbank.org/) and then using PPP to market exchange rates from OECD, taken from the IIASA SSP database (104). The maintenance costs are scaled with GDP projections per country taken from the same database.

These benefits and costs of mangrove restoration represent a first-order approximation of the economic case for restoration. To strive for greater realism in our analysis, we incorporate regrowth rates, and sensitivity to survival rate and discount rates. First, restored mangroves do not provide full ecosystem services immediately upon planting but rather develop their protective capacity gradually as they mature, with full benefits typically achieved only after years (44, 105, 106). Various studies note that maximum wave attenuation and coastal protection capacities are only reached after 5 to 10 y with rapid initial growth (44, 107, 108). Therefore, we include a temporal progression of benefit accrual with an exponential growth following Eq. **4** to reach saturation at 10 y.

[4] $$B=1-e^{-k(t-t_{0})}.$$

Second, mangrove restoration projects often experience significant mortality, with success rates typically ranging from 60 to 90% after 10 y based on available records, with some projects failing completely (43, 101). We further scale our benefits and maintenance costs with a survival rate of 75% and provide a sensitivity to survival rates of 50 and 90%. Last, the mangrove restoration costs from Goto et al. (100) are implementation costs and therefore do not include opportunity costs (foregone economic returns from alternative land uses), which would increase total restoration costs substantially. For example, Busch et al. (109) estimated that opportunity costs are 1.4 times the costs of implementation costs. To include opportunity costs, we calculated total costs as the sum of implementation costs plus opportunity costs (TC = IC + 1.4 × IC), where IC represents implementation costs from Goto et al. (100).

The total benefits and costs of mangrove restoration at present values were calculated for each year until 2100, summed, and discounted over time. BCR are estimated by dividing the total discounted benefits by the total discounted costs, following Eq. **4** and NPV following Eq. **5**.

[5] $$R_{C}^{B}=\frac{\sum_{t=1}^{n}\frac{B_{t}}{{\left(1+r\right)}^{t}}}{\left(\sum_{t=1}^{n}\frac{C_{t}}{{\left(1+r\right)}^{t}}\right)},$$

[6] $$V=\sum_{t=1}^{n}\frac{B_{t}-C_{t}}{{\left(1+r\right)}^{t}},$$

where $R_{C}^{B}$ is the benefit–cost ratio, $C_{t}$ the costs at time t, V the net present value, and r is the discount rate. The benefit–cost analysis is calculated for the benefits and costs of adaptation for subnational regions. These regions are defined as the administrative unit below national scale in the Global Administrative Areas Database (GADM). The benefits and costs are discounted using a discount rate of 5%, with sensitivity analyses conducted at 3, 8, and 10% to account for variation in appropriate discount rates across different economic contexts.

To calculate benefits, we assess assets in all areas that are hydraulically connected to the end point of transects (a 2D model), extending inland to include all potential flood-prone areas. Transects where there are no urban areas or where there is no simulated inundation (i.e., those not prone to flood risk) are excluded.

The analysis is undertaken for two different sea-level rise scenarios (RCPs) and five different socioeconomic scenarios (SSPs). Results are shown for two scenario combinations (110), namely RCP4.5/SSP2 and RCP8.5/SSP5. The former (RCP4.5/SSP2) is used for a “middle of the road” scenario with medium challenges and adaptation (104) that can broadly be aligned with the Paris agreement targets (111), while the latter is used as a “fossil-fuel development” world (112). Results of the other combinations are presented in *SI Appendix*.

### Wealth Disparity analysis.

In this study, we provide an assessment of the distributional impacts of mangrove restoration for two indicators: people living below a poverty line (113) and regional wealth index (114). We estimate these effects for three population groups, namely: i) populations living outside flood-prone areas; (ii) populations living in flood-prone areas; and iii) populations living in flood-prone areas who may benefit from mangrove restoration. For the first indicator—people living under a poverty line—three countries were selected where mangrove restoration is possible and gridded data of people living under a poverty line are available, namely Bangladesh, Nigeria, and Pakistan. For Bangladesh, we use the Bayesian-based geostatistics map from Steele et al. (115), using US$2.5 per day as the poverty line threshold. For Nigeria and Pakistan, the mean likelihood of people living in poverty was from Tatem et al. (116). The distributional impacts of mangrove restoration on population dynamics are assessed with the regional wealth index to estimate differences in population distributions per group and was done for 12 countries. Distributional curves of population frequency and wealth index for the three population groups were assessed based on microestimates of population regional wealth index by Chi et al. (114) for 2018 at the resolution of 2.4 km. For the Khulna region in Bangladesh, we use the regional wealth index estimates from Steele et al. (115) owing to its better coverage. We assess the effects on both indicators by using an overlay of the population living in the three areas with the likelihood of people living in poverty and the wealth index for each of the three countries. To test for differences between populations in the different areas, the Mann–Whitney U test is selected, as distributions were not Gaussian.

## Supplementary Material

Appendix 01 (PDF)

## Data Availability

Subnational data of flood risk benefits and benefit–cost analysis of mangrove restoration are stored on Zenodo (https://doi.org/10.5281/zenodo.17994385) (117). All other data are included in the article and/or *SI Appendix*.
