# Supplementary material for: Mangrove restoration and coastal flood adaptation: A global perspective on the potential for hybrid coastal defenses
Source: Proc Natl Acad Sci U S A. 2026 Jan 20;123(4):e2510980123. doi: 10.1073/pnas.2510980123 (PMC12846779; doi:10.1073/pnas.2510980123)
Supplement: Supplementary file 1 — Appendix 01 (PDF) [file pnas.2510980123.sapp.pdf]

PNAS

Supporting Information for
Mangrove restoration and coastal flood adaptation: a global perspective on the potential for hybrid coastal defences

Timothy Tiggeloven*1,2, Vincent van Zelst3,4, Eric Mortensen1, Bregje K van Wesenbeeck3,4 Thomas A Worthington5, Mark Spalding5,6, Hans de Moel1 & Philip J Ward1,3

1 Vrije Universiteit Amsterdam, Institute for Environmental Studies, De Boelelaan 1087, 1081 HV Amsterdam, Netherlands
2 CMCC Foundation - Euro-Mediterranean Center on Climate Change, Via della Libertà 12, Venice, 30175, Venice, Italy
3 Deltares, PO Box 177, NL2600MH Delft, the Netherlands
4 Delft University of Technology, Faculty of Civil Engineering and Geosciences, P.O. Box 5048, 2600 GA Delft, The Netherlands
5 University of Cambridge, Department of Plant Sciences, Cambridge, CB2 3EA, United Kingdom
6 The Nature Conservancy, Strada delle Tolfe 14, Siena 53100, Italy

*Correspondence to: Timothy Tiggeloven.
Email: timothy.tiggeloven@vu.nl

This PDF file includes:

Figures S1 to S3
Tables S1 to S2

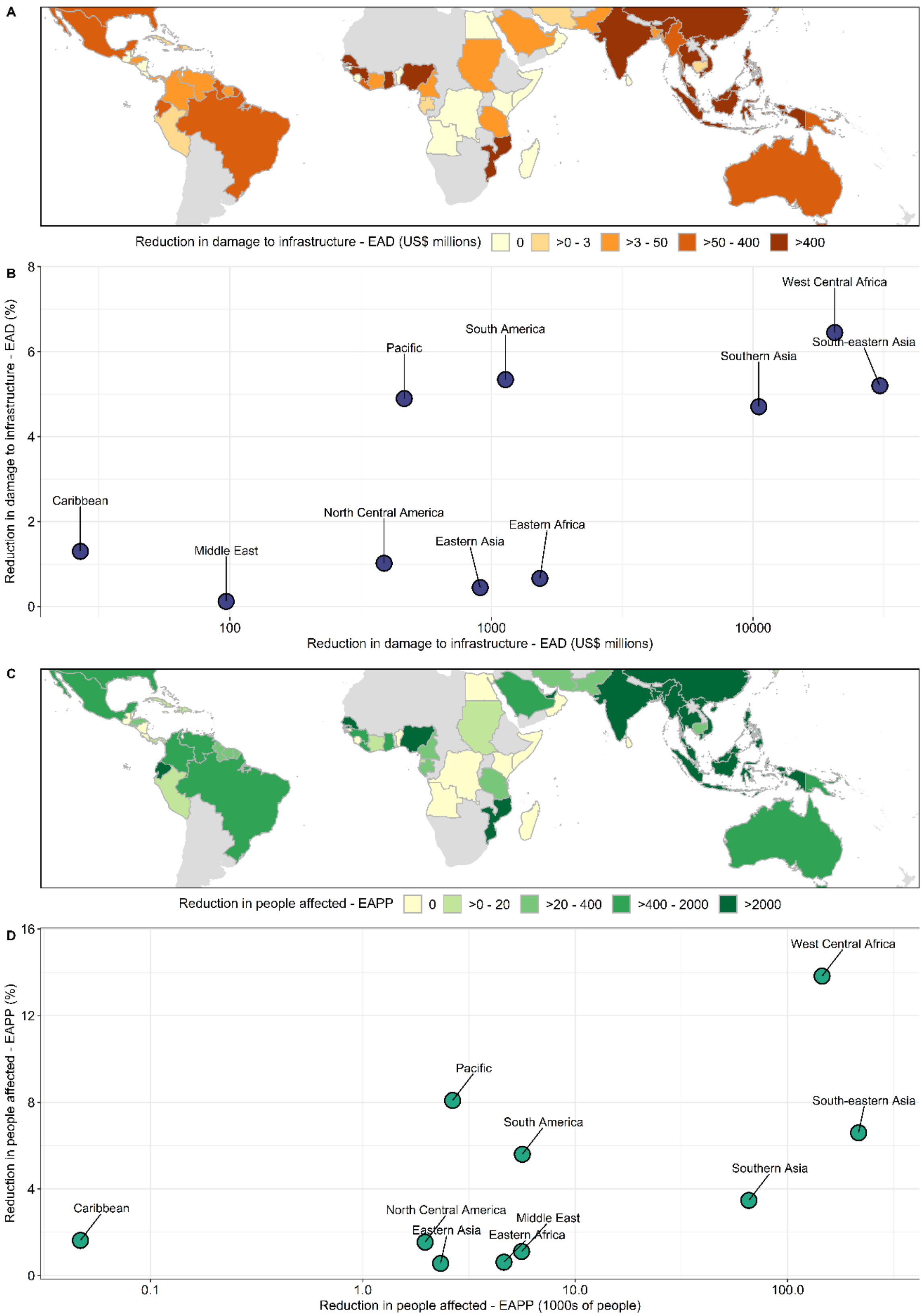
A
Reduction in damage to infrastructure - EAD (US$ millions)
0
>0 - 3
>3 - 50
>50 - 400
>400
B
Reduction in damage to infrastructure - EAD (%)
8
6
4
2
0
West Central Africa
South America
Pacific
South-eastern Asia
Southern Asia
Caribbean
North Central America
Eastern Africa
Eastern Asia
Middle East
100
1000
10000
Reduction in damage to infrastructure - EAD (US$ millions)
C
Reduction in people affected - EAPP
0
>0 - 20
>20 - 400
>400 - 2000
>2000
D
Reduction in people affected - EAPP (%)
16
12
8
4
0
West Central Africa
Pacific
South-eastern Asia
South America
Southern Asia
Caribbean
North Central America
Middle East
Eastern Asia
Eastern Africa
0.1
1.0
10.0
100.0
Reduction in people affected - EAPP (1000s of people)

Fig. S1. Future (2080) expected annual damage reduction in damage to built-up assets (2a and 2b) and people affected (2c and 2d) for countries (2a and 2c) and supra-national regions (2b and 2d) under the scenarios RCP8.5/SSP5.s

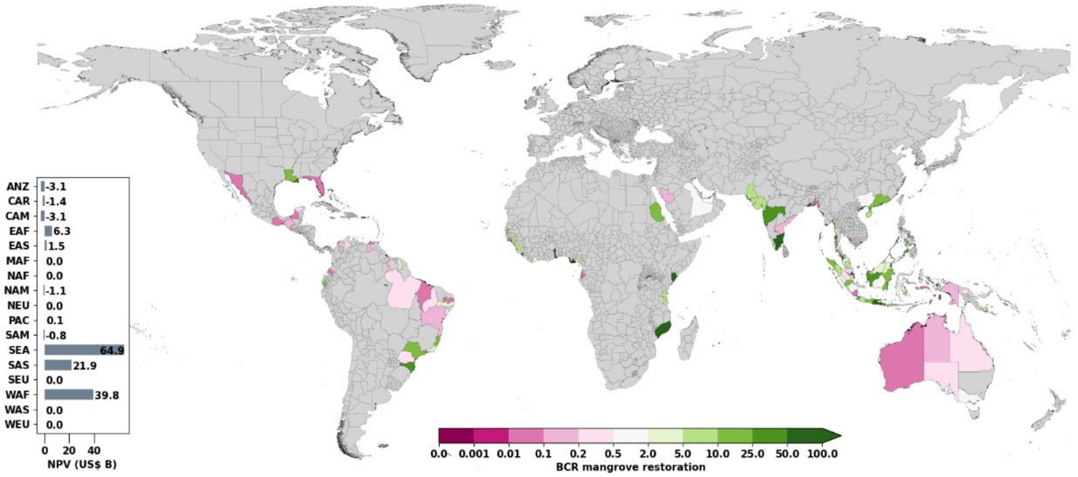

Fig. S2. Benefit-Cost Ratios of mangrove restoration under the future scenario of RCP8.5/SSP5 shown for sub-national regions in the world plot and sub-continental regions in the subplot. ANZ, Australia and New Zealand; CAR, Caribbean; CAM, Central America; EAF, Eastern Africa; EAS, Eastern Asia; MAF, Middle Africa; NAF, Northern Africa; NEU, Northern Europe; PAC, Pacific regions that include Melanesia, Polynesia, and Micronesia; SAM, South America; SEA, South-eastern Asia; SAS, Southern Asia; WAF, Western Africa; WAS, Western Asia; WEU, Western Europe.

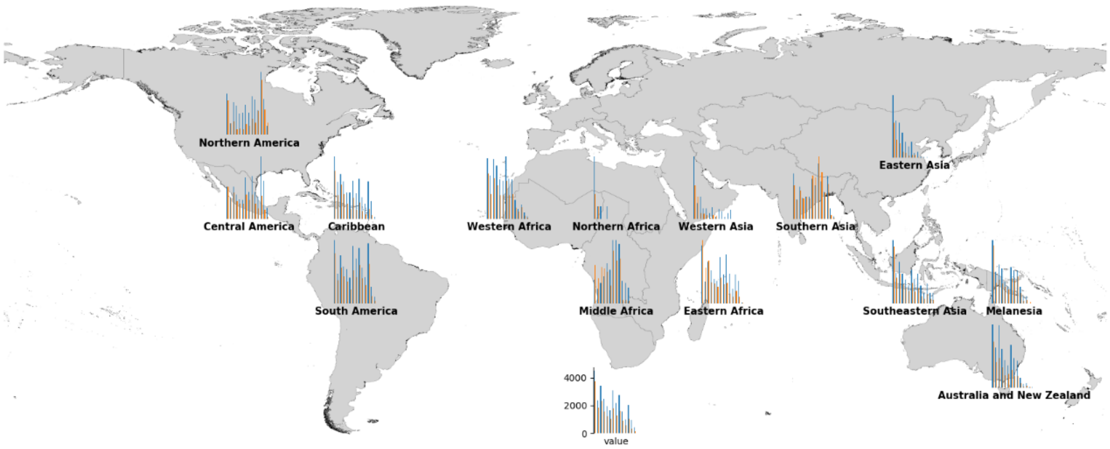

Fig. S3. Distribution of restorable mangrove forest width per urban/rural transects.

Table S1. Country scale ranking of the (future) benefits of mangrove restoration in terms of flood risk reduction under present-day conditions and future scenarios. EAD and NPV values are displayed in US$ billion and EAPE in thousands.

Present-day				RCP4.5/SSP2						RCP8.5/SSP5					
**EAD**		**EAPE**		**EAD**		**EAPE**		**NPV**		**EAD**		**EAPE**		**NPV**	
**ISO**	**Value**	**ISO**	**Value**	**ISO**	**Value**	**ISO**	**Value**	**ISO**	**Value**	**ISO**	**Value**	**ISO**	**Value**	**ISO**	**Value**
NGA	0,205	NGA	35,6	NGA	5,6	NGA	150,3	VNM	14.4	NGA	17.0	NGA	139.6	VNM	35.8
IND	0,166	IDN	33,0	VNM	4,5	VNM	97,8	NGA	12.5	VNM	13.4	VNM	95.0	NGA	30.8
IDN	0,157	IND	20,8	IDN	4,3	PHL	75,9	IND	9.2	IDN	11.9	IND	61.3	IDN	24.2
MYS	0,061	PHL	17,0	IND	3,8	IND	57,2	IDN	9.1	IND	10.5	PHL	59.8	IND	22
USA	0,056	VNM	13,8	PHL	1,1	IDN	53,4	GIN	3.5	PHL	2.6	IDN	49.3	MOZ	5.9
VNM	0,029	MMR	2,7	GIN	0,9	MOZ	5,4	MOZ	2.4	GIN	1.8	MYS	5.5	GIN	5.7
CHN	0,026	MYS	2,2	MYS	0,6	SEN	5,2	PHL	1.2	MOZ	1.5	ARE	5.1	PHL	2.6
AUS	0,023	MOZ	2,2	MOZ	0,5	MYS	5,1	CHN	0.7	MYS	1.3	MOZ	4.4	MYS	1.5
PHL	0,016	CHN	2,1	THA	0,4	ARE	4,2	SEN	0.6	THA	1.0	THA	4.1	CHN	1.4
BRA	0,013	THA	1,9	CHN	0,4	BGD	4,2	GHA	0.5	CHN	0.9	BGD	4.0	SEN	1.2
GUF	0,009	BGD	1,7	SEN	0,4	THA	3,5	MYS	0.4	SEN	0.7	CHN	2.3	GHA	1.1
ECU	0,008	SEN	1,6	GHA	0,2	GIN	2,5	ECU	0.2	GHA	0.6	MMR	2.1	LBR	0.9
MOZ	0,007	BRA	1,3	AUS	0,2	MMR	2,5	MMR	0.2	LBR	0.4	SEN	2.0	MMR	0.7
GHA	0,006	ECU	0,8	USA	0,2	CHN	2,1	LBR	0.2	AUS	0.4	ECU	2.0	ECU	0.6
MMR	0,005	GUY	0,5	GUF	0,1	BRA	2,0	TZA	0.1	GUF	0.4	BRA	1.9	GUF	0.4

Table S2. Sensitivity of global and regional BCR and NPV results to discount rate, survival rate, and exclusion to opportunity cost assumptions under RCP4.5 and RCP8.5. World additionally reports the number of regions with BCR > 1.

Region	Indicator	rcp4p5							rcp8p5						
		Baseline	Discount 3%	Discount 8%	Discount 10%	Survival 50%	Survival 90%	No opp. costs	Baseline	Discount 3%	Discount 8%	Discount 10%	Survival 50%	Survival 90%	No opp. costs
World	BCR	2.76	5.28	1.24	0.83	2	3.16	4.98	5.97	12.6	2.23	1.33	4.32	6.85	10.78
	NPV	43.62	129.9	4.96	-3.59	22.74	56.15	54.72	124.61	355.72	26.45	6.47	76.67	153.37	135.81
	Regions	85	102	65	57	76	89	100	105	124	73	63	96	108	118
Australia and New Zealand	BCR	0.16	0.31	0.07	0.05	0.11	0.18	0.29	0.23	0.5	0.09	0.06	0.17	0.27	0.43
	NPV	-3.43	-3.38	-3.32	-3.23	-3.35	-3.47	-1.56	-3.12	-2.44	-3.25	-3.2	-3.15	-3.11	-1.26
Caribbean	BCR	0.01	0.03	0	0	0.01	0.01	0.02	0.03	0.08	0.01	0	0.02	0.04	0.06
	NPV	-1.47	-1.69	-1.31	-1.25	-1.37	-1.52	-0.78	-1.44	-1.61	-1.3	-1.24	-1.35	-1.49	-0.75
Central America	BCR	0.04	0.09	0.02	0.01	0.03	0.05	0.08	0.07	0.15	0.02	0.01	0.05	0.08	0.13
	NPV	-2.93	-3.37	-2.63	-2.51	-2.75	-3.04	-1.54	-3.08	-3.37	-2.81	-2.7	-2.91	-3.18	-1.58
Eastern Africa	BCR	42	54.08	27.85	21.87	32.57	46.48	63.4	103	145.66	55.62	37.62	79.88	113.99	155.48
	NPV	2.5	5.07	1.19	0.83	1.66	3.01	2.52	6.23	13.81	2.42	1.46	4.14	7.48	6.25
Eastern Asia	BCR	5.11	11.84	1.63	0.85	3.68	5.88	9.4	9.64	24.61	2.56	1.17	6.93	11.09	17.73
	NPV	0.67	2.08	0.09	-0.02	0.41	0.83	0.75	1.41	4.54	0.22	0.02	0.9	1.72	1.49
Melanesia	BCR	0.86	1.6	0.36	0.22	0.64	0.97	1.42	1.95	4.07	0.64	0.33	1.46	2.2	3.21
	NPV	-0.01	0.06	-0.04	-0.05	-0.02	0	0.02	0.07	0.33	-0.02	-0.04	0.03	0.1	0.11
Middle Africa	BCR	0.28	0.61	0.1	0.06	0.21	0.33	0.51	0.83	1.92	0.25	0.12	0.6	0.95	1.49
	NPV	-0.06	-0.04	-0.06	-0.06	-0.06	-0.06	-0.02	-0.01	0.09	-0.05	-0.06	-0.03	0	0.02
Northern America	BCR	0.61	0.92	0.37	0.29	0.43	0.7	1.16	0.68	1.07	0.41	0.32	0.48	0.79	1.3
	NPV	-1.12	-0.43	-1.49	-1.58	-1.42	-0.94	0.06	-1.14	-0.22	-1.56	-1.65	-1.43	-0.96	0.04
South America	BCR	0.38	0.81	0.15	0.1	0.28	0.44	0.71	0.74	1.71	0.24	0.13	0.53	0.85	1.37
	NPV	-1.93	-0.72	-2.33	-2.37	-2.11	-1.82	-0.48	-0.82	2.65	-2.09	-2.27	-1.37	-0.49	0.62
Southeastern Asia	BCR	4.13	7.33	2.02	1.41	3.01	4.72	7.29	9.06	17.69	3.78	2.38	6.61	10.35	16
	NPV	25.18	63.38	6.99	2.62	14.8	31.41	28.67	64.86	167.29	19.01	8.91	41.25	79.02	68.34
Southern Asia	BCR	6.64	12.08	3.02	2.03	4.94	7.5	11.08	14.73	29.49	5.43	3.12	10.97	16.64	24.58
	NPV	8.98	23.64	2.61	1.24	5.63	11	9.62	21.87	60.77	5.73	2.55	14.22	26.47	22.51
	BCR	43.1	72.87	18.75	11.61	33.26	47.82	65.85	98.04	181.7	35.04	18.6	75.64	108.78	149.78

**Western Africa**	**NPV**	17.25	45.16	5.35	2.9	11.42	20.75	17.39	39.76	113.54	10.27	4.81	26.43	47.76	39.9
**Western Asia**	**BCR**	0.86	1.54	0.42	0.3	0.62	0.99	1.56	1.07	2.28	0.41	0.25	0.78	1.23	1.95
	**NPV**	-0.03	0.13	-0.1	-0.12	-0.07	0	0.06	0.02	0.33	-0.11	-0.13	-0.04	0.05	0.11
